# On the Proximate Causes of Chronic Ophthalmia

**Published:** 1830-07

**Authors:** William John Thomas


THE LONDON
### Medical and Physical Journal.

No. 377, VOL. LXIV.] JULY 1830. [No. 49, *New Series.*

For many fortunate discoveries in medicine, and for the detection of numerous errors, the world is indebted to the rapid circulation of Monthly Journals; and there never existed any work to which the Faculty, in Europe and America, were under deeper obligations than to the *Medical and Physical Journal of London*, now forming a long but an invaluable series.—*Rush.*

### ORIGINAL PAPERS, AND CASES,
### OBTAINED FROM PUBLIC INSTITUTIONS AND OTHER AUTHENTIC SOURCES.

### CHRONIC OPHTHALMIA.

### *On the Proximate Causes of Chronic Ophthalmia.*

By WILLIAM JOHN THOMAS, M.R.C.S.

THERE is a species of indolent inflammation of the cornea, which we frequently meet with in practice, of a very stubborn and intractable nature, occasioning severe suffering to the patient, and frequently much embarrassment to the practitioner. I desire to offer a few remarks upon this disease: I am induced to do so from having lately met with several cases of this description, which yielded within a moderate time to the exhibition of appropriate remedies.

Inflammations of the eye, of an acute nature, generally arise from those causes which induce similar affections in other organs; but there are specific agents to which, from its exposed situation, the eye is more peculiarly obnoxious: to enumerate these would occasion unnecessary prolixity in the present communication, which I intend to render as brief and concise as the nature of the subject will admit. The form of inflammation which I am now considering may be materially modified in its manifestations by the idiosyncrasy of the individual. I lately had a person under my care who had chronic inflammation of the cornea, of several years' duration, and whose prevailing diathesis appeared to be the rheumatic: the affection was considerably alleviated by the administration of colchicum, combined with calomel and opium; the local disease subsiding under the alteration of the constitutional action. In the suppression of local affections, too much attention can scarcely be paid to the state of the constitution. I am persuaded, however, that, in many diseases of particular organs, and more especially of the eye, the irritative sympathy of contiguity will be found to influence materially the progress of the morbid phenomena; and, although a remote or sympathetic irritant may have called the primary disease into action, yet the consecutive changes in the phenomena thus produced will vary with the intensity of action and proximity of the local irritation.

In the course of this paper, I shall have occasion to advert to some particular examples of this nature, and shall for the present proceed to consider the succession of morbid phenomena which is exhibited during the progress of this disease. The primary symptoms are either of an acute or subacute nature: in the scrofulous diathesis, the latter generally prevails. If the acute form exist, the symptoms are, of course, of a more aggravated nature; the congestion of the sanguiferous vessels more conspicuous, and the epiphora and intolerance of light more troublesome. After these symptoms have existed for an indefinite period, the state of chronic inflammation becomes permanently established, and the nature of the exciting cause will be found to have changed, as the phenomena exhibited in the chronic cases are varying from those of the acute. The primary proximate causes of the acute inflammation may be either idiopathic or sympathetic irritation. This irritation first excites the diseased action, and subsequently accelerates it, accompanying the inflammatory action to the complete destruction of the diseased organ.

When the inflammation is of the passive kind, the capillary congestion in many cases proves an exciting cause to the morbid action of the congested vessels. The physical distention is a stimulus to the arterial tubes, and one of the sources of the prolonged irritability of the eye. It is curious to trace the chain of these phenomena, of the varying operations of the causes and effects, and of the several morbid changes resulting from their combined actions. Let us consider the dilatation of the arterial calibers of the more superficial vessels, the sympathetic irritation which it necessarily excites, and the preternatural actions dependent upon that irritation. The transparent vessels, when distended with red blood, become considerably congested; they occupy certain spaces which were appropriated to the reception of other vessels, and the reticulated cellular tissue is lacerated in consequence of their displacement. Upon the first admission of arterial blood in larger quantities into these vessels, the irritability of their parietes is necessarily augmented. The physical distention of the containing organs is the next phenomenon resulting from the increased momentum of the scarlet stream. When the arterial tubes are first excited to action, the power of resistance may be nearly equivalent to the momentum of impression. The successive waves of blood, acted upon by the *vis à tergo* and by the specific action excited in the radical branches by the stimulus of congestion, augment the impelling powers. This may be called the active inflammatory stage. At this period, the pain is acute and pungent; the secretions are hot and acrid; the tears more especially offending the irritable organ, and excoriating the adjacent parts. These symptoms continue for an indefinite time; their nature changes as the phenomena vary from the acute stage: the irritability of the organ begins to subside.

The cause of this effect is worthy of consideration. We have before observed that, at the first invasion of the disease, the additional quantity of blood proves a stimulus to the substance of the arterial tubes. This stimulus is that specific principle of irritability resident in the blood: whether this be a chemical or a galvanic agent, it is not necessary in this place to inquire. It may be expedient, however, to observe that this principle of irritability is attracted by the parietes of the living arteries, and communicates, by its specific stimulus, the susceptibility which they possess. The ingress of the blood is rapid, but its egress from the tube by no means equivalent to the primary invasion.

A physical distention of the organ necessarily ensues; the blood, being detained in its transit, imparts to the beating artery a much greater portion of its specific stimulus than is consistent with its own vitality. This life-imparting fluid dies, in the very act of furnishing to the excited organ the final minimum of its principle of irritability. Having expended this, it becomes chemically decomposed, and the fibrine, adhering to the internal parietes, intercepts for a time the acquisition of the vital stimulus by the walls of the artery, from the current of blood which now passes with little impediment through the tube. The resistance of irritability being thus overcome in the vessel, the power of elasticity subsides also, and the only obstruction offered to the laceration of the tube exists in the mechanical power of resistance, resident in the substantial density of the arterial walls. The contracting principle of reaction having withdrawn from the vessels, the physical distention may be completely established, and the tonic powers of the artery permanently withdrawn: that portion of the tube primarily affected, as well as the capillary apertion which terminates its ramification, will become paralytic.

The fibrine will now be urged slowly through the barriers of coagulating lymph into the reticulated texture of the cellular tissue; whilst nature, opposed in her first endeavours to impede the destructive progress of the disease, fortifies, with fibrinous laminæ, the walls of the artery above, and blockades the capillary termination of the tube with a cone of coagulated lymph. Thus terminates the active stage of the inflammation.

The fibrine effused into the cellular tissue, and between the laminæ of the cornea, retains its primary aspect until condensed and consolidated into complete opacity, by the absorption of its fluid parts. If no proximate exciting cause have arisen, the action of the organic trains subsides into quiescent tranquillity. The only traces of the acute inflammation are opacities and ulcerations of the cornea. The former may, by the judicious administration of scientific remedies, be ultimately removed; and the latter, by appropriate applications, be in a great measure restored to their primary convexity.

It frequently happens that the phenomena of inflammation, especially in the severely acute species from local injury, subside very tardily, or not at all: we may then suspect that some local and exciting cause has supervened on the original injury. I believe it will be found that, the more intense the degree of the superficial irritation, the more likely the chronic stage will be to follow in succession.

The conjunctiva is the external covering of the eye; the same tunic covers the ball and the interior of the palpebræ. These, therefore, will frequently be found to react upon each other, and to sympathise in their respective inflammations. There are three states in which the palpebræ are frequently found: the first that of simple arterial vascularity. In this stage of inflammation the pain is trifling, and the symptoms are comparatively mild. The appearance of the disease may be compared to a patchwork of scarlet velvet on a strawcoloured ground of a smoother quality. It is seldom that a solitary insulated portion is perceptible; for several of these distinct figures of vascularity may be seen on the first inversion of the lids, immediately before the stimulus of the mechanical displacement causes the blood to fill the minute vessels of the membrane, producing, as a necessary consequence, the temporary congestion of the tunic of the ciliary cartilage.

These insulated portions of arterial vascularity are then the first stage of that peculiar series of phenomena which introduces the proximate and exciting causes of chronic ophthalmia. The activity of the inflammation in these insulated portions will be observed to be accompanied with deposits of fibrine; detached fragments of which frequently float towards the internal canthus. These foreign agents increase the inflammatory action, and portions of this coagulating lymph attached to the inflamed patches become successively organized. The organization of these fibrinous bodies has something peculiar in its arrangement. False membranes are not detected, as in the inflammations of the peritoneum, &c.; but the newly-formed vessels, instead of ramifying over the extended surface of the fibrine, instinctively tend towards each other, and, by their own elective affinities, condense the inflammatory process within a circumscribed capacity. The lymph having now become vascular, assumes an organic appearance: the organization of this substance exhibits an uneven aspect, with alternate elevations and depressions. The depressions, in the progress of the disease, are rapidly excavated, and the original elevations being increased by the intense vascularity of the accuminated processes of these newly-formed tubercles, the whole of the conjunctiva originally affected is transformed into a series of granulating patches. These detached portions subsequently coalesce, the circumferences of their basements uniting in every direction, and forming new centres of preternatural actions, accelerating the progress of this destructive disease. During this stage, ulcerations of the cornea proceed with rapidity, from the acuminated papillæ of the granulating surfaces stimulating the cornea to destructive inflammation.

In the third and last stage, the granulations assume a fungoid appearance; they are no longer granulated tubercles, but appear like vast carneous masses interspersed with deep and penetrating clefts. The fungoid tumors are not rounded off, but form angular projections. Frequently we perceive a number of these masses crowded together; they appear, indeed, as one solid mass, on a superficial inspection, but upon minutely examining them, especially by separating them with a probe, we detect the real nature of their constitution. They may be compared to lobes, with flat surfaces opposed to each other; processes of a delicate membrane, apparently of a serous nature, covering their opposing surfaces, and secreting a fine transparent fluid, avert that friction of their several aspects which might lead to an adhesive and consolidating inflammation between their surfaces.

A person lately put himself under my care with fungoid excrescences of the conjunctiva: he is blind; the cornea of both eyes are opaque, in part from solid interstitial deposits, of a circumscribed nature, and partly from effused lymph of a more superficial character. The disease has existed twelve months. The apparently central deposits, I strongly suspect, are preceded by interstitial absorption, or inflammatory ulceration. It is somewhat difficult to conceive, considering the extreme density of the texture connecting and consolidating the laminæ of the adult cornea, in which way these albuminous patches have primarily originated. If we consider them as simple fibrinous effusions, what a force must have projected the lymph so as to deposit it in the condensed connexions of the corneal laminæ! If, however, the interstitial absorption precedes the deposit, we can readily perceive how the effused lymph may occupy the space excavated by the hand of nature for its reception. In this light these opaque deposits may be esteemed the results of a salutary process of nature, to unload the adjacent vessels, and to relieve the patient from the intolerable pain produced by the congested vessels, tearing asunder the condensed laminæ of the transparent cornea. These opaque spots which occupy the central departments of the cornea are therefore the results of the inflammatory action of vessels pervading the interior tunics of the laminated transparency. In the natural state, these vessels are diaphanous, and carry a pellucid fluid, eliminated from the blood: they are principally prolongations of the superficial vessels, partaking of their peculiar character. If we review the progress of this disease in its different stages, we perceive the preternatural formations causing the greatest inconvenience. Towards the third stage, however, the cornea, in the majority of cases which have come under my observation, appears insensible to the stimulus of the fungoid excrescences, being itself completely opaque, and partially disorganized.

Having thus endeavoured to point out a fertile source of embarrassment to those practitioners who are not aware of the frequency of its occurrence, I must express my belief of the high improbability of the successful treatment of chronic ophthalmia, if these exciting causes are overlooked.

When the disease has not produced disorganization of the cornea, the interference of the art of surgery may suspend its progress, and avert that unhappy termination. The proximate exciting cause must be first removed, and the subsequent treatment directed to the effects which have already resulted from the specific irritant. By perseverance in the practice of these principles, we may frequently restore the despairing patient to the inestimable blessings of the light of heaven, to his own great comfort and satisfaction, to the public credit and benefit of the successful practitioner, and to the permanent honour of the art of surgery.

*Liverpool; June 2d,* 1830.

---

